# Out-of-plane polarization induces a picosecond photoresponse in rhombohedral stacked bilayer WSe_2_

**DOI:** 10.3762/bjnano.15.109

**Published:** 2024-11-06

**Authors:** Guixian Liu, Yufan Wang, Zhoujuan Xu, Zhouxiaosong Zeng, Lanyu Huang, Cuihuan Ge, Xiao Wang

**Affiliations:** 1 Key Laboratory for Micro-Nano Physics and Technology of Hunan Province, College of Materials Science and Engineering, School of Physics and Electronics, Hunan University, Changsha, 410082, China

**Keywords:** broken inversion symmetry, out-of-plane polarization, picosecond photoresponse, time-resolved photocurrent measurement (TRPC), WSe_2_

## Abstract

Constructing van der Waals materials with spontaneous out-of-plane polarization through interlayer engineering expands the family of two-dimensional ferroelectrics and provides an excellent platform for enhancing the photoelectric conversion efficiency. Here, we reveal the effect of spontaneous polarization on ultrafast carrier dynamics in rhombohedral stacked bilayer WSe_2_. Using precise stacking techniques, a 3R WSe_2_-based vertical heterojunction was successfully constructed and confirmed by polarization-resolved second harmonic generation measurements. Through output characteristics and the scanning photocurrent map under zero bias, we reveal a non-zero short-circuit current in the graphene/3R WSe_2_/graphene heterojunction region, demonstrating the bulk photovoltaic effect. Furthermore, the out-of-plane polarization enables the 3R WSe_2_ heterojunction region to achieve an ultrafast intrinsic photoresponse time of approximately 3 ps. The ultrafast response time remains consistent across varying detection powers, demonstrating environmental stability and highlighting the potential in optoelectronic applications. Our study presents an effective strategy for enhancing the response time of photodetectors.

## Introduction

Two-dimensional (2D) van der Waals (vdW) ferroelectrics with dangling-bond-free surfaces exhibit ferroelectricity by effectively resisting depolarization fields, providing a promising platform for highly integrated devices [1–3]. The emergence of ferroelectricity at the atomic scale in vdW ferroelectrics has garnered significant interest because of its potential applications in various fields [4–14]. Through interlayer engineering, it is viable to fabricate the 2D ferroelectrics using vdW materials, even when the monolayer exhibits centrosymmetry [15–20]. Two-dimensional ferroelectrics constructed through interlayer engineering typically break out-of-plane (OOP) symmetry, resulting in the spontaneous OOP polarization. Currently, the spontaneous OOP polarization has been confirmed in several materials, such as AB-stacked h-BN [16–17,21], WTe_2_ [22], 1T' ReS_2_ [23], bilayer MoS_2_ directly exfoliated from 3R-MoS_2_ bulk crystal [24], and artificially stacked bilayers transition metal dichalcogenides (TMDs) through twist misalignment [18–19], which brings about fascinating physical phenomena [25–29].

Stacking semiconductor vdW materials with suitable bandgaps at specific angles not only breaks OOP symmetry, but also combines excellent semiconductor properties with spontaneous polarization, offering promising advances in optoelectronics [23,30]. One of the key optoelectronic phenomena in 2D semiconductor materials is the photocurrent response. The polarization, which results in spontaneous photocurrent under zero bias, gives rise to the bulk photovoltaic effect (BPVE), which can lead to a high-efficiency photoelectric conversion that has the potential to surpasses the Shockley–Queisser limit [24,31–34]. In this regard, constructing 2D vdW semiconductors with OOP polarization and moderate bandgap holds great promise for high-performance self-powered BPVE devices. More importantly, spontaneous polarization can further modulate the photogenerated carriers, which provides the potential for self-powered switchable and ultrafast photodetectors [35–37]. However, the impact of polarization effects on photogenerated carrier dynamics in these artificially stacked materials remains unexplored.

In this work, we confirmed the spontaneous OOP polarization in artificially stacked 3R WSe_2_ and investigated the ultrafast carrier dynamics through time-resolved photocurrent (TRPC) measurements. We fabricated 3R WSe_2_ through artificial stacking in parallel and validated the broken inversion symmetry by second harmonic generation (SHG) measurements. The broken symmetry in 3R WSe_2_ leads to the BPVE. To confirm the BPVE, a vertical heterojunction of graphene-wrapped bilayer 3R WSe_2_ was fabricated. The non-zero short-circuit current was observed in the output characteristics and the scanning photocurrent map under zero bias. In addition, the OOP polarization accelerates the drift of photogenerated carriers, giving the heterojunction region an ultrafast intrinsic response time of approximately 3 ps, surpassing that of graphene under the same conditions. Despite variations in probe power, the ultrafast response time remains consistent, highlighting the reliability of the high-speed heterojunction photodetector across different conditions, confirming its potential application in the field of optoelectronics. Our findings enhance the comprehension of the impact of polarization on photogenerated carriers and have the potential to advance related device applications.

## Results and Discussion

### Out-of-plane polarization of 3R WSe_2_

**Figure 1:**
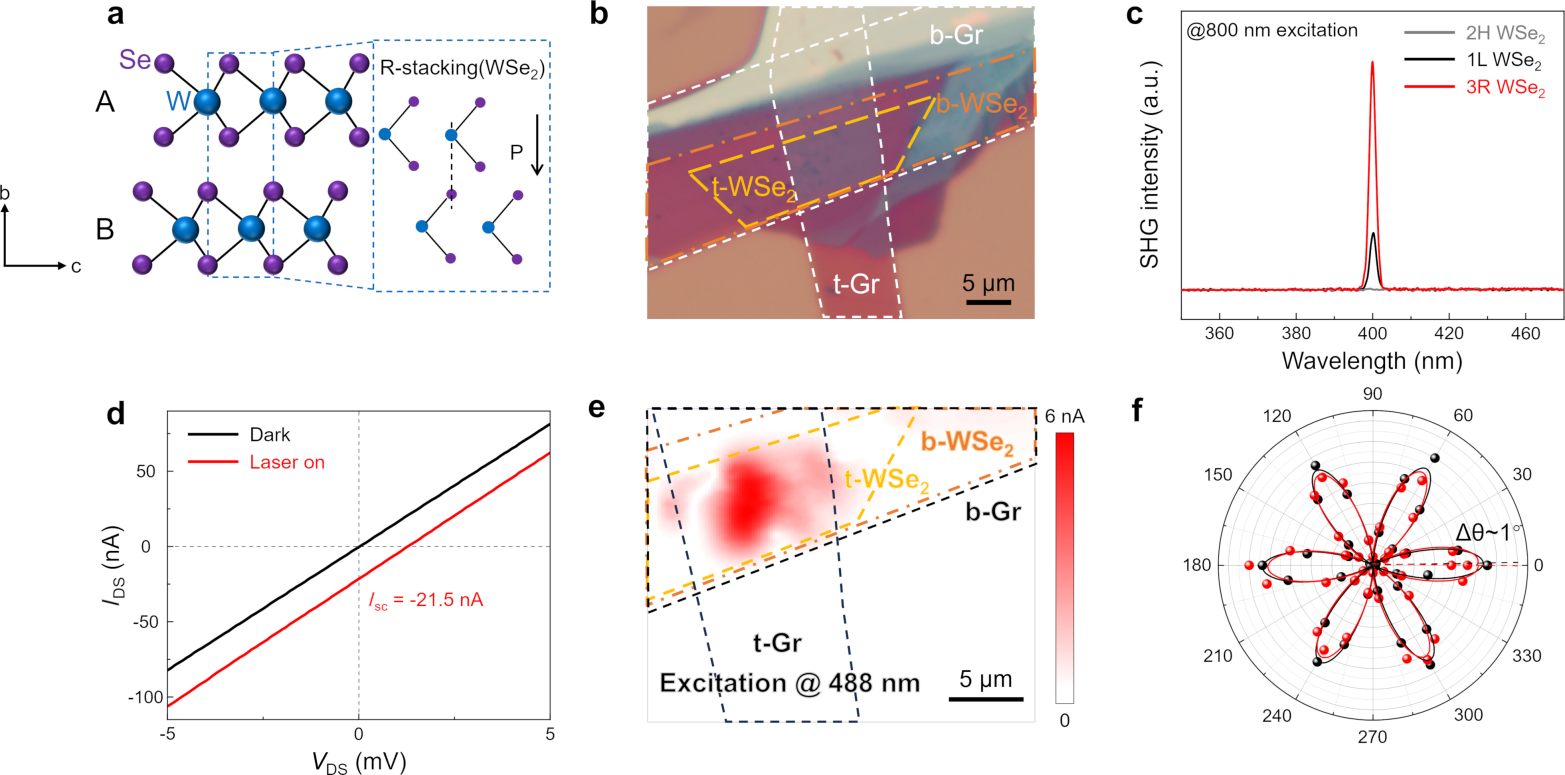
Crystal structure and the OOP polarization of 3R WSe_2_. (a) Crystal structure of 3R bilayer WSe_2_. The blue and purple spheres, respectively, represent W and Se atoms. The black arrow represents the direction of interlayer polarization. (b) Optical image of the graphene/3R WSe_2_/graphene heterojunction device. The yellow and orange dashed lines represent two monolayers of WSe_2_. The white dashed lines represent the top and bottom graphene layers. (c) SHG spectra of H-stacked (2H) and R-stacked (3R) bilayer WSe_2_. (d) Output characteristic curves of the device shown in (b) under dark (black) and illuminated conditions (λ = 520 nm, red). Under 520 nm laser illumination, there is a significant short-circuit current of about 21.5 nA. (e) Scanning photocurrent microscopy map obtained with an excitation wavelength of 780 nm on the device in (b) with the *V*_ds_ = 0 V and *V*_g_ = 0 V. The yellow and orange dashed lines indicate two monolayers of WSe_2_ stacked in parallel. The black dashed lines represent the top and bottom graphene layers. (f) Polarization-resolved SHG signals of top (black dots) and bottom (red dots) WSe_2_ monolayers.

Precisely controlling the stacking angle of two monolayers in TMDs can modify the symmetry and induce unique physical properties. Depending on the stacking order, bilayer WSe_2_ can be divided into 3-rhombohedral (3R) and 2-hexagonal (2H) phases [24,26,38]. When two layers are stacked antiparallel, bilayer WSe_2_ exhibits a hexagonal stacked (H-stacked) structure with inversion symmetry. In contrast, artificially stacking two layers in parallel to form a rhombohedral stacked (R-stacked) structure (as illustrated in Figure 1a) disrupts the OOP mirror symmetry. Because of differences in the stacking order, 3R WSe_2_ can exist in two forms, AB and BA, which determine the polarization direction (see Supporting Information File 1, Figure S1). We define the bilayer WSe_2_ stacking depicted in Figure 1a as AB, where the tungsten atoms (W, blue dots) are positioned directly above the selenium atoms (Se, purple dots). This arrangement leads to charge transfer from the lower layer to the upper layer, resulting in downward polarization [24] (as shown by the black arrow in Figure 1a). In contrast, the BA stacking order, opposite to AB, induces upward polarization (Figure S1a). The transition between AB and BA stacking orders can be accomplished through the interlayer sliding of adjacent WSe_2_ layers.

To further investigate the OOP polarization in 3R WSe_2_, we prepared the graphene/3R bilayer WSe_2_/graphene vertical heterojunction shown in Figure 1b. We performed SHG measurements with an 800 nm laser to excite monolayer WSe_2_ and artificially stacked bilayer WSe_2_. A stronger SHG intensity at 400 nm was detected in bilayer WSe_2_ constructed by artificial stacking, which confirmed its 3R phase (Figure 1c). To highlight the difference in SHG intensity between bilayer WSe_2_ of various phases and monolayer WSe_2_, we controlled the stacking angle to prepare 3R and 2H regions on the same monolayer WSe_2_ (Supporting Information File 1, Figure S2a). The corresponding SHG scanning map is presented in Supporting Information File 1, Figure S2b. Subsequently, polarization-resolved SHG measurements were conducted on the top and bottom WSe_2_ regions in Figure 1b. The deviation between the two crystal orientations was approximately 1°, confirming the high precision of the stacking angle. To enrich the basic characterizations of WSe_2_, we conducted Raman spectroscopy and scanning electron microscopy (SEM) measurements (Supporting Information File 1, Note 5).

The broken symmetry leads to an asymmetric distribution of photogenerated carriers, resulting in a non-zero photocurrent even under zero bias, known as BPVE [33,39]. Under 520 nm laser excitation, a short-circuit current of approximately 21.5 nA was observed in the output characteristic curve of the vertical heterojunction device (Figure 1d), proving the existence of spontaneous OOP polarization. To compare to the graphene/WSe_2_/graphene devices, we fabricated a WSe_2_ two-terminal device, the electronic characteristics of which are shown in Supporting Information File 1, Note 4. To utilize the BPVE in 3R WSe_2_, we investigated the vertical heterojunction using scanning photocurrent microscopy (SPCM). The scanning photocurrent map obtained under zero bias (Figure 1e), demonstrates that the photocurrent mainly originates from the heterojunction region, thus, further confirming the OOP polarization. These measurements of the non-zero short-circuit current in the heterojunction region provide a basis for subsequent research on internal carrier dynamics.

### Ultrafast photoresponse in the vertical graphene/3R WSe_2_/graphene heterojunction

**Figure 2:**
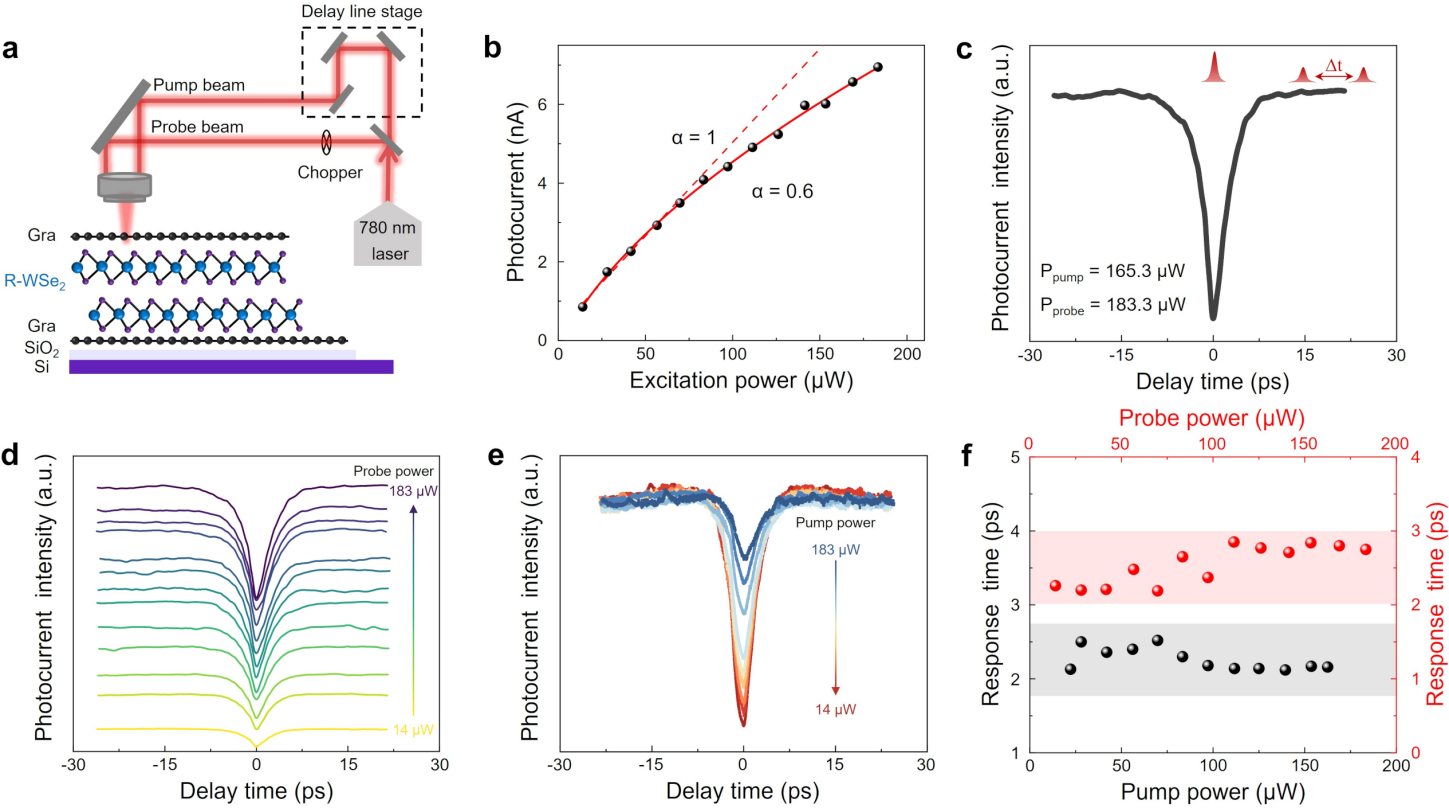
TRPC measurements in the graphene/3R WSe_2_/graphene heterojunction. (a) Schematic of the graphene/3R WSe_2_/graphene device and the TRPC setup. (b) Sublinear dependence of photocurrent on excitation power, indicating the saturation effect. A power law fit was applied, resulting in *I*_ph_ ≈ *P*^0.6^. (c) Typical TRPC results of the graphene/3R WSe_2_/graphene device. The intrinsic photoresponse time of 2.8 ps was obtained through exponential fitting. (d) Probe power-resolved TRPC curves for the graphene/3R WSe₂/graphene heterojunction device, ranging from 14 to 183 μW. (e) Pump power-resolved TRPC curves for the graphene/3R WSe₂/graphene heterojunction device, ranging from 14 to 183 μW. (f) Photoresponse time (τ) of the heterojunction as a function of pump (black dots) and probe power (red dots).

Theoretically, the OOP polarization in 3R WSe_2_ accelerates the separation of photogenerated carriers, suggesting that the vertical heterojunction may exhibit a fast photoresponse. We explored the photogenerated carrier dynamics and the intrinsic photoresponse in the vertical heterojunction through time-resolved photocurrent measurements (TRPC). The custom-built TRPC setup is shown in Figure 2a. A 780 nm pulse laser is used as the excitation source and split into two separate beams, one serving as the pump and the other as the probe. The two beams focus spatially on the same point within the graphene/3R WSe_2_/graphene heterojunction region. The measurements are based on the conventional pump–probe method, requiring the pump power to have sufficiently high power to saturate the sample [40–42].

Figure 2b shows the power-law relationship between photocurrent (*I*_ph_) and excitation power (*P*), *I*_ph_ = *P*^0.6^, indicating that the photocurrent exhibits sublinear growth, consistent with the saturation effect. In Figure 2c, the TRPC measurement curve shows a symmetric dip at zero time delay, attributed to the significant suppression due to ground state saturation. By fitting the TRPC curve with an exponential function, we determined that the photoresponse time of the heterojunction region is approximately 2.8 ps. The picosecond photoresponse in the heterojunction region arises not only from the ultrashort channel length in the vertical direction but also from the OOP polarization of WSe_2_. In 3R WSe_2_, polarization accumulates with increasing layer thickness, resulting in a higher polarization value in thicker multilayered 3R WSe_2_ nanoflakes, which will accelerate the separation of photogenerated carriers. Subsequently, we investigated the TRPC curves of the heterojunction region across a range of probe and pump powers, ranging from 14 to 183 µW (Figure 2d,e). Each TRPC curve has been individually fitted with an exponential function to obtain the intrinsic photoresponse time (τ), as summarized in Figure 2f. The response time (τ) of the device can be determined by fitting the time-resolved photocurrent signals with the following equation [40–41,43]:


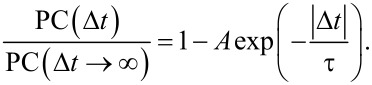


Here, the amplitude *A* and the time constant τ are the fitting parameters. With different probe and pump powers, the ultrafast response time remains consistent, demonstrating that the high-speed heterojunction photodetector can work under various conditions.

### Comparison of TRPC in the heterojunction and graphene regions

**Figure 3:**
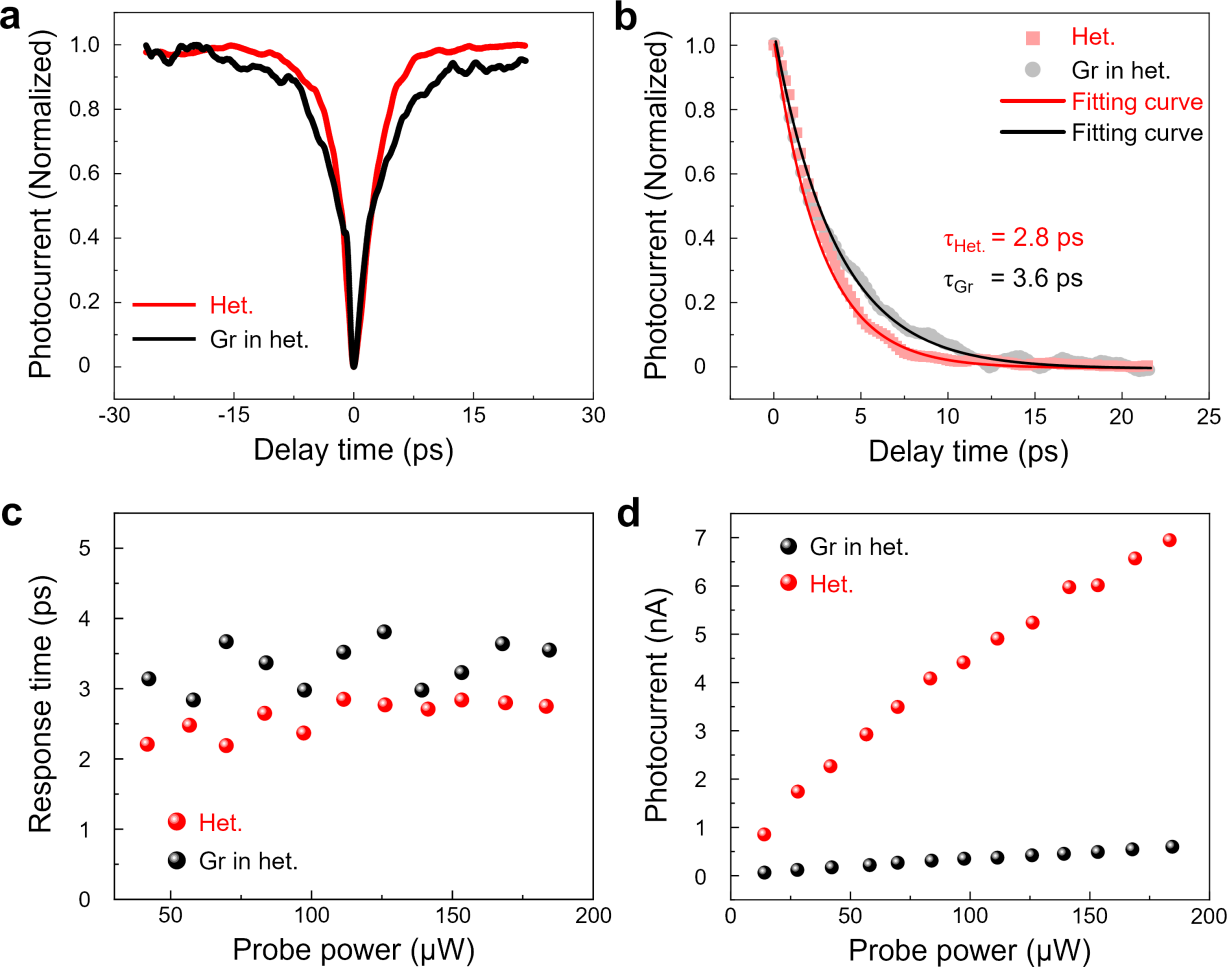
(a) Comparison of TRPC curves in the heterojunction and in the graphene region. (b) Normalized photocurrent decay curves of the heterojunction and graphene in the heterojunction. The solid fitting curves have been fitted by exponential decay. (c) Photoresponse time (τ) of heterojunction (red dots) and graphene region (black dots) as a function of probe power. (d) Photoresponse intensity of heterojunction (red dots) and graphene region (black dots) as a function of probe power.

We then considered the intrinsic response time of graphene within the heterojunction. Because of its high carrier mobility and broadband absorption, graphene has been used to prepare high-speed photodetectors with intrinsic photocurrent response times down to the picosecond level [42]. Notably, under the same excitation conditions, comparison of the TRPC curves between the heterojunction and the graphene region in the heterojunction revealed that the heterojunction exhibits an even shorter response time (Figure 3a,b). It is the OOP polarization within the bilayer WSe_2_ that accelerates the intrinsic response time of the heterojunction. To verify the reliability of the experimental results, we subsequently conducted TRPC measurements at various probe powers in the heterojunction and in the graphene region, with the power ranging from 40 to 184 µW. The response times of the heterojunction (red dots) and graphene region (black dots) are summarized in Figure 3c. The response time of the heterojunction is consistently shorter than that of the graphene region. This finding not only confirms the stability of the device but also demonstrates the acceleration effect of OOP polarization in 3R WSe_2_ on carrier drift. Although the power change does not affect the response time of the device, the increase in power generates more photogenerated carriers, reflected in the increased magnitude of the photocurrent (Figure 3d).

## Conclusion

We confirmed that the photodetector based on 3R phase WSe_2_ exhibits an ultrafast photocurrent response on the picosecond scale. The 3R bilayer WSe_2_ was artificially fabricated by precisely controlling the stacking angle of two monolayers of WSe_2_. The crystal structures of 3R WSe_2_ leads to the breaking of OOP symmetry, confirmed by SHG measurements. Subsequently, the bulk photovoltaic effect in the graphene/3R WSe_2_/graphene vertical heterojunction was confirmed through the output characteristic curve and photocurrent map under zero bias. The bulk photovoltaic response time is approximately 3 ps. Because of the spontaneous OOP polarization, the response time of the heterojunction is even shorter than that of graphene under the same conditions, which highlights its potential application in ultrafast photoelectric detection.

## Experimental

### Device fabrication

The 2D materials, WSe_2_ and graphene, were obtained from high-quality bulk crystals using the mechanical exfoliation method. The monolayer WSe_2_ nanoflakes were exfoliated onto a transparent polydimethylsiloxane (PDMS) film, selected based on optical contrast, and characterized by photoluminescence (PL) measurements at room temperature (see Supporting Information File 1, Figure S3). The relative angles between crystalline axes of the top and bottom WSe_2_ layers were determined by polarization-resolved SHG measurements. The zigzag directions of the two monolayers of WSe_2_ were aligned at a 0° angle to form the 3R phase. The graphene/3R WSe_2_/graphene heterojunctions were aligned and assembled onto a SiO_2_/Si substrate by the all-dry transfer method. Au/Cr (50/10 nm) electrodes were patterned using standard electron-beam lithography (EBL, Raith 150 Two) and deposited onto the heterojunctions through metal thermal evaporation.

### Material and performance characterization

The optical images of heterojunctions were obtained using a microscope (ZEISS, Axio Scope A1). The SHG and PL measurements were performed by using a scanning confocal microscope (WITec, alpha 300R) at room temperature. The electrical properties of the devices were characterized using an Agilent B1500 semiconductor analyzer in a Lake Shore vacuum chamber (10^−4^ Pa).

### SPCM and TRPC measurement

The photocurrent maps were all obtained under zero bias using a custom-built scanning photocurrent microscopy (SPCM) with a 780 nm fiber laser (NPI Rainbow 780 OEM) mechanical chopped at 1050 Hz. The laser was focused near the diffraction limit on the samples by an objective (Olympus LMPLFLN 50×). The photocurrent was collected by a lock-in amplifier (Stanford SR830) with a background noise of approximately 0.2 pA. Photocurrent maps were generated by scanning the desired area using a piezoelectric displacement stage equipped with a fixed laser spot. The scanning range covered a maximum square area of 100 µm.

Using time-resolved photocurrent measurements (TRPC), we explored the intrinsic photoresponse time and carrier dynamics. The TRPC setup involves two separate beams of 780 nm ultrashort laser pulses with 80 fs pulse width. Both laser beams were focused on the same point on the samples. The probe beam is chopped by a mechanical chopper at 1050 Hz. The delay time between the two beams is precisely controlled by a mechanical delay stage (Thorlabs DDSM100/M). The pump and probe beams were recombined using a beam splitter following the delay line stage. Pump and probe beams simultaneously excite the sample at zero delay. With increasing delay, the excited carriers relax before being re-excited by the probe, weakening the photocurrent suppression. This results in an exponential recovery with a response time τ, causing a typical symmetric dip in the TRPC curve at zero delay.

## Supporting Information

File 1 Characterization of structure, SHG image, SEM and EDS images, Raman and PL spectrum of WSe_2_ and raw TRPC curves for the extraction of response time.

## Data Availability

The data that supports the findings of this study is available from the corresponding author upon reasonable request.
