# Supplementary material for: Out-of-plane polarization induces a picosecond photoresponse in rhombohedral stacked bilayer WSe2
Source: Beilstein J Nanotechnol. 2024 Nov 6;15:1362–8. doi: 10.3762/bjnano.15.109 (PMC11552432; doi:10.3762/bjnano.15.109)
Supplement: File 1 — Characterization of structure, SHG image, SEM and EDS images, Raman and PL spectrum of WSe2 and raw TRPC curves for the extraction of response time. [file Beilstein_J_Nanotechnol-15-1362-s001.pdf]

BEILSTEIN JOURNAL OF NANOTECHNOLOGY

Supporting Information

for

Out-of-plane polarization induces a picosecond photoresponse in rhombohedral stacked bilayer WSe_2

Guixian Liu, Yufan Wang, Zhoujuan Xu, Zhouxiaosong Zeng, Lanyu Huang, Cuihuan Ge and Xiao Wang

Beilstein J. Nanotechnol. **2024,** *15,* 1362–1368. doi:10.3762/bjnano.15.109

Characterization of structure, SHG image, SEM and EDS images, Raman and PL spectrum of WSe_2 and raw TRPC curves for the extraction of response time

License and Terms: This is a supporting information file under the terms of the Creative Commons Attribution License (https://creativecommons.org/licenses/by/4.0). Please note that the reuse, redistribution and reproduction in particular requires that the author(s) and source are credited and that individual graphics may be subject to special legal provisions.
The license is subject to the *Beilstein Journal of Nanotechnology* terms and conditions: (https://www.beilstein-journals.org/bjnano/terms)

Table of Contents:

Note 1: Side view of crystal structure in bilayer WSe_2.

Note 2: Direct comparison of SHG intensity between 3R and 2H bilayer WSe_2.

Note 3: Confirmation of monolayer WSe_2 by PL spectrum.

Note 4: Electronic characteristics of the WSe_2 two-terminal device.

Note 5: Basic characterizations of WSe_2.

Note 6: TRPC curves of the graphene/3R WSe_2/graphene heterojunction and graphene in heterojunction at different probe powers.

Note 1: Side view of crystal structure in bilayer WSe_2.

The crystal structure of 3R bilayer WSe_2 with AB stacking is given in the main text (Figure 1a). When the 3R bilayer WSe_2 with BA stacking order, the selenium atoms (Se, purple dots) are directly above the tungsten atoms (W, blue dots), leading to upward spontaneous polarization (Figure S1a). The AB and BA stacking order result in opposite polarization directions [1]. In contrast, the zigzag directions of the two monolayer WSe_2 are aligned at an angle of 180° (or 60°) [2,3], forming the 2H phase with spatial inversion symmetry (Figure S1b). Therefore, there is no out-of-plane spontaneous polarization in 2H WSe_2 [4]. The difference in symmetry between 3R and 2H WSe_2 is clearly evident from their crystal structures.

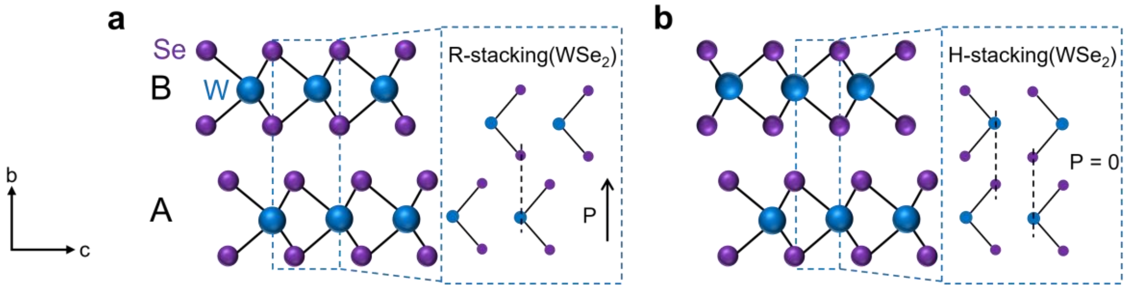

Figure S1: Side views of crystal structure in bilayer WSe_2. a 3R phase with BA stacking order. **b** 2H bilayer WSe_2.

Note 2: Direct comparison of SHG intensity between 3R and 2H bilayer WSe_2.

To clearly compare the symmetry differences between the 3R and 2H phases, we simultaneously recorded the SHG intensity maps of bilayer WSe_2 for both phases. First, we stacked an additional monolayer of WSe_2 onto the same monolayer at 0° and 180°, respectively, to construct the 3R and 2H phases (optical image is shown in Figure S2a). The entire area containing the 3R and 2H bilayer WSe_2 was scanned with 800 nm pulsed

laser, and the resulting SHG map is shown in Figure S2b. The difference in SHG intensity among the single layer, 3R bilayer, and 2H bilayer WSe_2 is clearly observed in the SHG image, consistent with Figure 2c in the main text.

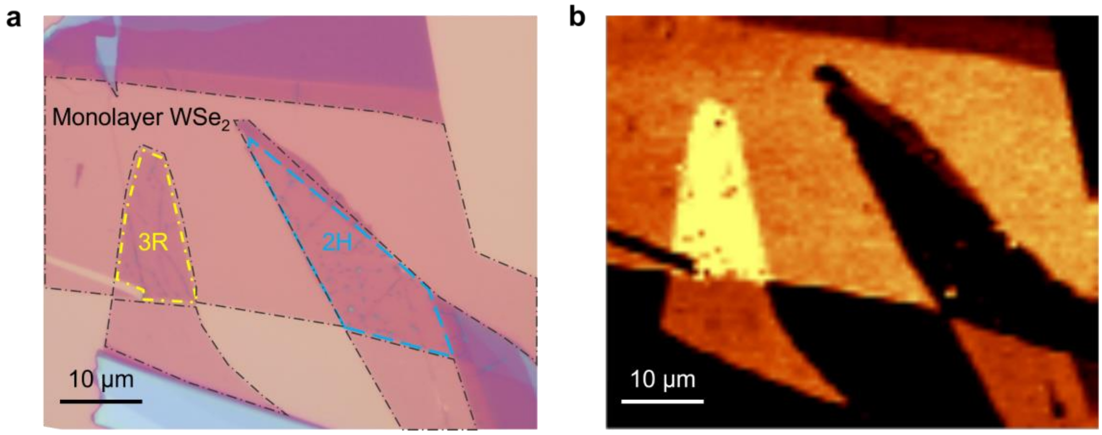

Figure S2: a Optical image of artificially stacked 3R and 2H bilayer WSe_2 **b** Corresponding SHG image.

Note 3: Confirmation of monolayer WSe_2 by PL spectrum.

Monolayer WSe_2 exhibits a direct band gap, leading to strong photoluminescence, in contrast to few-layer to bulk WSe_2, which is considered to have an indirect band gap [5,6]. Hence, the PL spectrum can be used to ascertain the monolayer nature of mechanically exfoliated WSe_2, facilitating the subsequent artificial stacking of 3R WSe_2. The peak position of PL for a monolayer WSe_2 is around 750 nm.

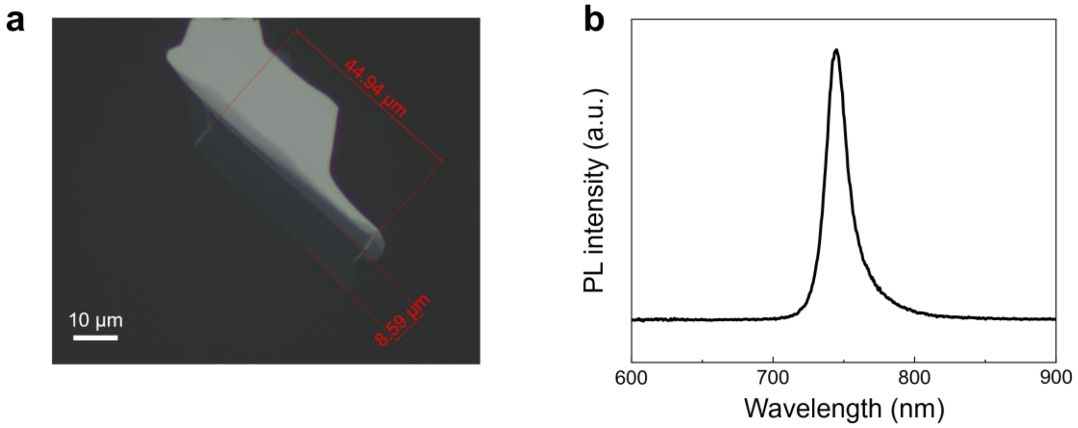

Figure S3: a Optical image of monolayer WSe_2 **b** Corresponding PL spectra at room temperature.

Note 4: Electronic characteristics of the WSe_2 two-terminal device.

We fabricated a WSe_2 two-terminal device to compare the graphene/WSe_2/graphene devices. The two-terminal device is constructed with WSe_2 as the channel material, a dielectric layer of approximately 290 nm SiO_2, and two parallel metal electrodes, composed of 10 nm Cr and 50 nm Au, functioning as the source and drain. The output characteristic curve I_{ds}–V_{ds} in the device is shown in Figure S4a. The transfer characteristic curves I_{ds}–V_g show an ambipolar channel conductance in WSe_2 (Figure S4b).

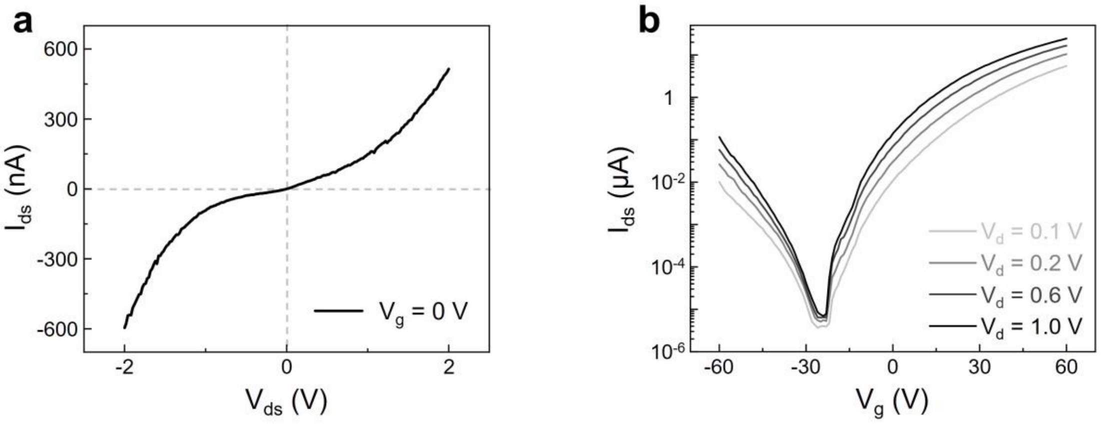

Figure S4: Electronic characteristics of the WSe_2 two-terminal device. a Output characteristic curves I_{ds}–V_{ds} at V_g = 0V. **b** Transfer characteristic curves I_{ds}–V_g with source-drain voltages varying from 0.1 V to 1.0 V on the semi-logarithmic scale.

Note 5: Basic characterizations of WSe_2.

To confirm the quality of the materials used, we performed Raman and scanning electron microscopy (SEM) measurements.

The Raman spectrum was obtained using a 532 nm continuous-wave laser as the excitation source. For the monolayer (1L) WSe_2, the spectra showed the characteristic peak of WSe_2 peak at 260 cm^{-1} (2LA(M)), as shown in Figure S5.

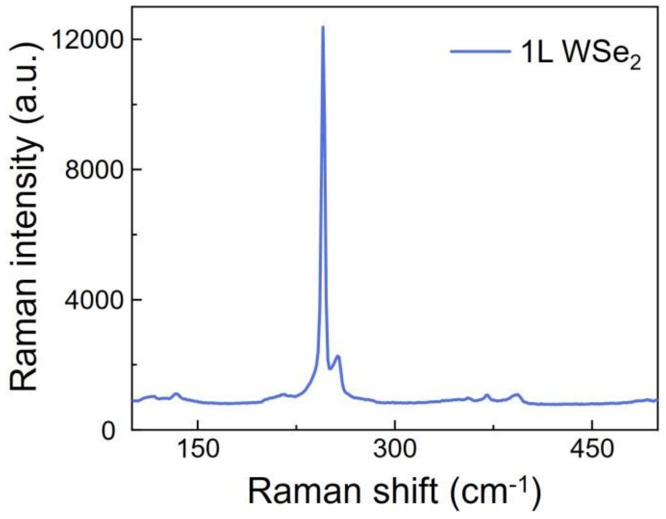

Figure S5: Raman spectrum of the monolayer WSe_2.

Scanning electron microscopy (SEM) measurements were performed on WSe_2 on a 290 nm SiO_2/Si substrate. The elemental mapping of the energy-dispersive spectroscopy (EDS) analysis (Figure S6c) confirmed tungsten (W) and selenium (Se) in the material.

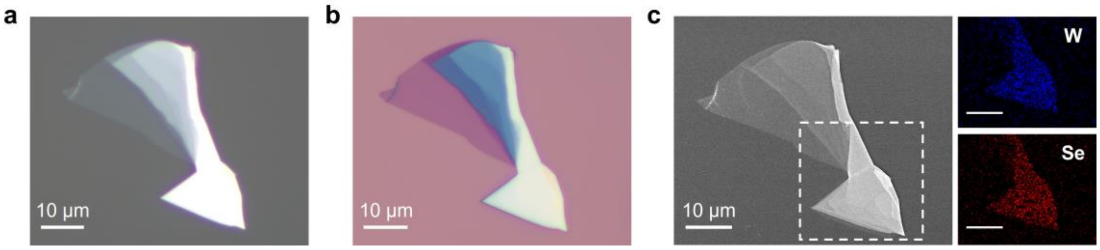

Figure S6: Scanning electron microscopy (SEM) measurements of WSe_2. **a** Optical image of the mechanically exfoliated WSe_2 on poly-dimethylsiloxane films (PDMS) **b** Optical image of the transferred the WSe_2 on silicon substrate. **c** Top view SEM and EDS image of WSe_2 on silicon substrate.

Note 6: Probe power dependent TRPC measurements of the graphene/ 3R WSe_2/graphene heterojunction and graphene in heterojunction.

In the main text, we compared the probe power dependent intrinsic response time of the heterojunction with that of graphene, as summarized in Figure 3c. We individually displayed the TRPC curves corresponding to each response time in Figure 3c of the main text. Figure S7 (graphene/3R WSe_2/graphene heterojunction) and S8 (graphene in heterojunction) show the TRPC curves at different probe powers, ranging from 42 μW to 184 μW. The TRPC curves for both the graphene and heterojunction regions remain essentially unchanged with varying probe power.

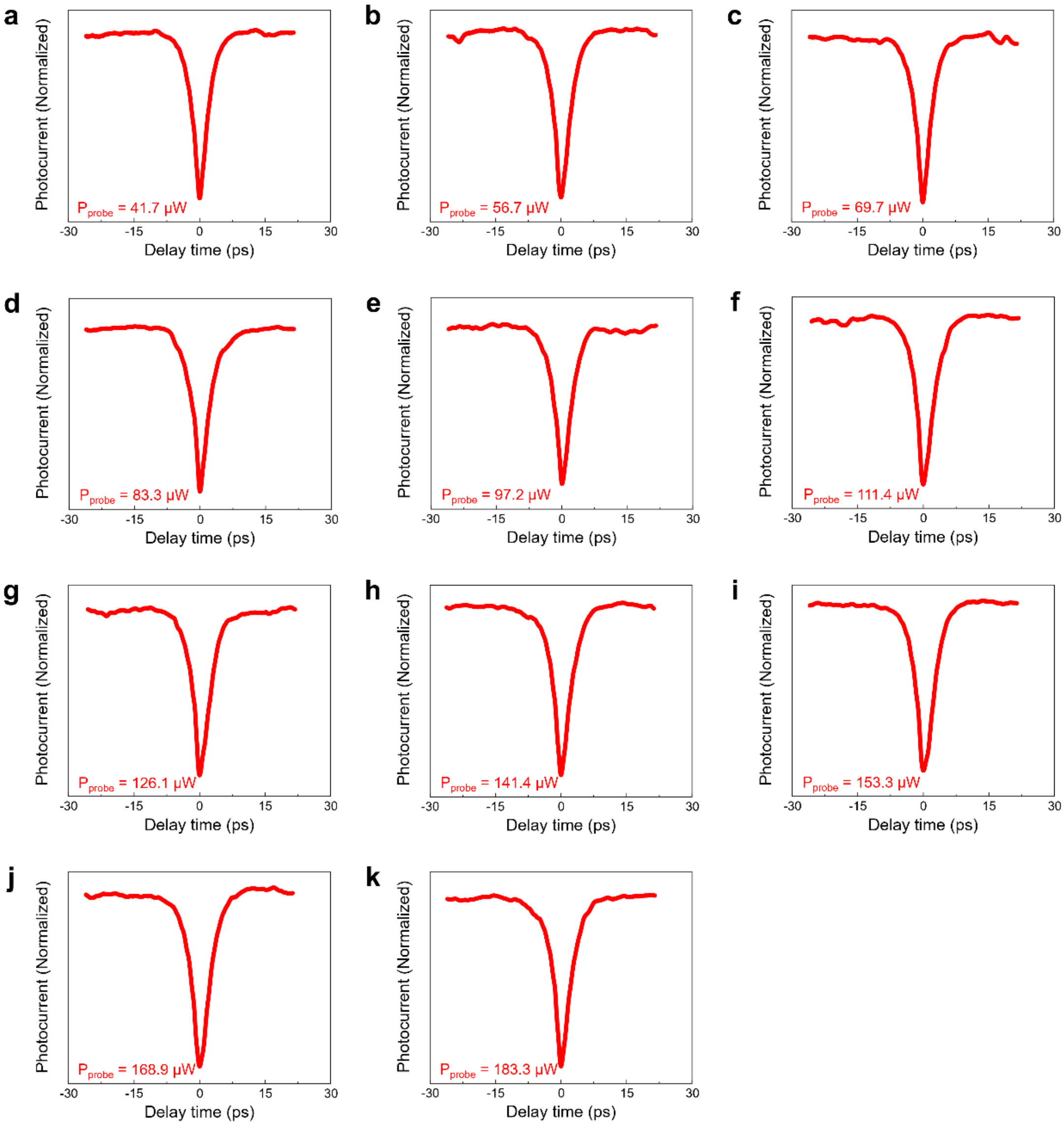

Figure S7.: TRPC curves of the graphene/3R WSe_2/graphene heterojunction at different probe powers, ranging from 41.7 μW to 183.3 μW.

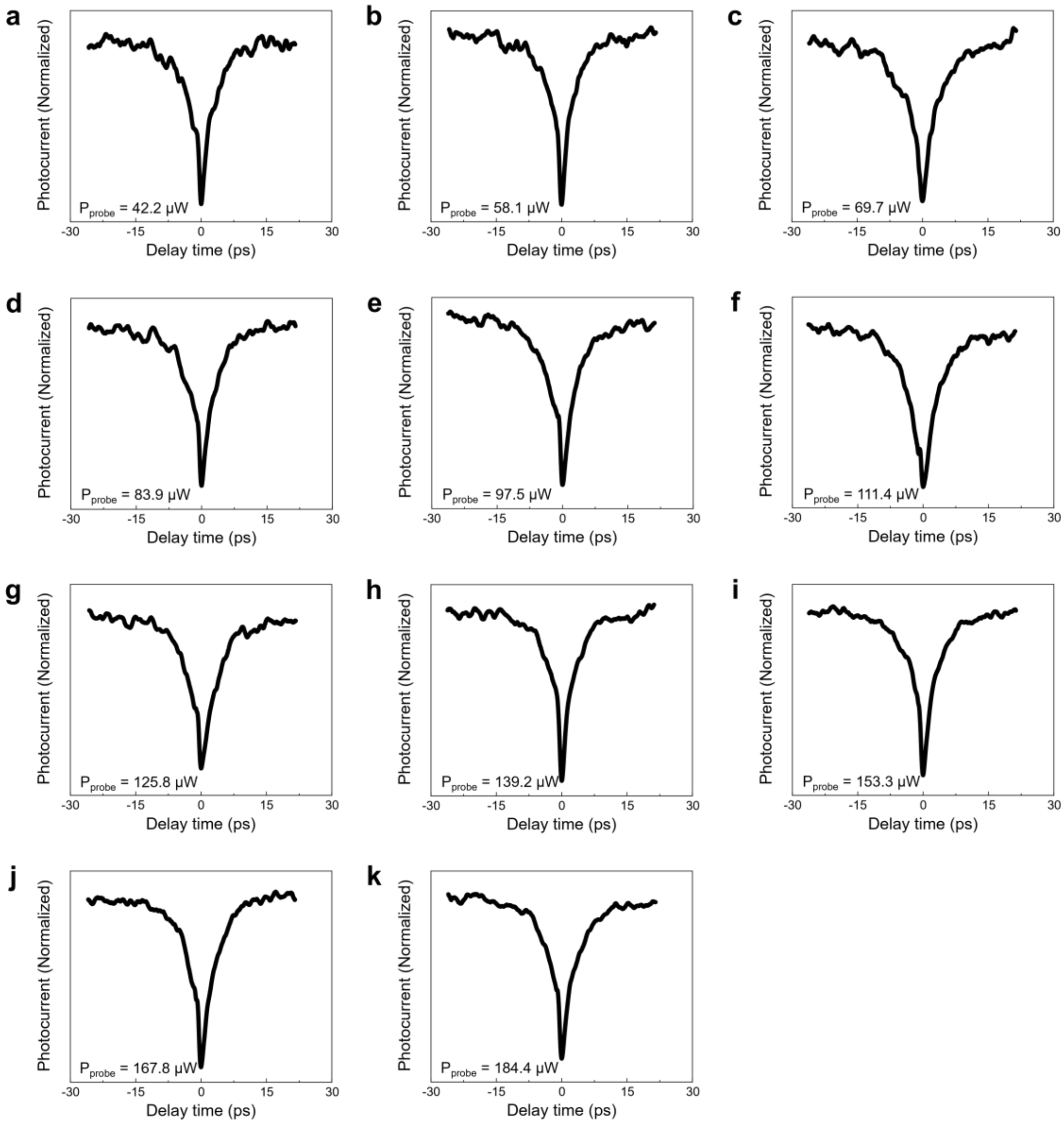

Figure S8: TRPC curves of the graphene in heterojunction at different probe powers, ranging from 42.2 μW to 184.4 μW.

Reference

[1] Wang, X.;Yasuda, K.;Zhang, Y.;Liu, S.;Watanabe, K.;Taniguchi, T.;Hone, J.;Fu, L. Jarillo-Herrero, P., *Nat. Nanotechnol.* **2022**, 17, 367.

[2] Ge, C.;Zhang, D.;Xiao, F.;Zhao, H.;He, M.;Huang, L.;Hou, S.;Tong, Q.;Pan, A. Wang, X., *ACS Nano* **2023**, 17, 16115.

[3] Ge, C.;Huang, L.;Zhang, D.;Tong, Q.;Zhu, X.;Wang, X. Pan, A., *AIP Adv.* **2023**, 13, 115101.

[4] Yang, D.;Wu, J.;Zhou, B. T.;Liang, J.;Ideue, T.;Siu, T.;Awan, K. M.;Watanabe, K.;Taniguchi, T.;Iwasa, Y.;Franz, M. Ye, Z., *Nat. Photonics* **2022**, 16, 469.

[5] Mak, K. F. Shan, J., *Nat. Photonics* **2016**, 10, 216.

[6] Xia, F.;Wang, H.;Xiao, D.;Dubey, M. Ramasubramaniam, A., *Nat. Photonics* **2014**, 8, 899.
